# Risk Factors and Relationship of Cutaneous and Uveal Melanocytic Lesions in Monozygotic and Dizygotic Twin Pairs

**DOI:** 10.1371/journal.pone.0160146

**Published:** 2016-08-03

**Authors:** Renáta Zsanett Csoma, Edit Tóth-Molnár, Anita Varga, Hajnalka Szabó, Hajnalka Orvos, Lajos Kemény, Judit Oláh

**Affiliations:** 1 Department of Dermatology and Allergology, University of Szeged, Szeged, Hungary; 2 Department of Ophthalmology, University of Szeged, Szeged, Hungary; 3 Department of Paediatrics, University of Szeged, Szeged, Hungary; 4 Department of Obstetrics and Gynaecology, University of Szeged, Szeged, Hungary; 5 Dermatological Research Group of the Hungarian Academy of Sciences, University of Szeged, Szeged, Hungary; Universidade de Sao Paulo, BRAZIL

## Abstract

**Background:**

The similar genetic background of a pair of twins, and the similar environmental impacts to which they are exposed allow an exact and objective investigation of various constitutional and environmental factors in naevus development. As far as we are aware, this is the first published survey that simultaneously examines cutaneous and ocular pigmented lesions in an appreciable sample of identical and non-identical twins.

**Methods:**

172 pairs of twins of Caucasian origin were included in this study. A whole-body skin examination and a detailed ophthalmological examination were performed to determine the density of melanocytic lesions. A standardized questionnaire was used to assess the data relating to constitutional, sun exposure and other variables.

**Results:**

A notably high proportion of the subjects (36.78%) manifested one or more clinically atypical melanocytic naevi (CAMNs), and approximately one-third (31.4%) of them at least one benign uveal pigmented lesion (BUPL). The incidence of iris freckles (IFs), iris naevi (INs) and choroidal naevi (CHNs) proved to be 25.35%, 5.98% and 3.52%, respectively. The interclass correlation coefficients for common melanocytic naevi (CMNs), CAMNs, and INs were 0.77, 0.76 and 0.86 in monozygotic twins, as compared with 0.5, 0.27 and 0.25 in dizygotic twin pairs, respectively. A statistically significant correlation was found between the prevalence of CAMNs and that of INs.

**Conclusions:**

This significant correlation suggests the existence of a subgroup of Caucasian people with an increased susceptibility to both cutaneous and ocular naevus formation. There is accumulating evidence that, besides the presence of cutaneous atypical naevi, INs can serve as a marker of a predisposed phenotype at risk of uveal melanoma. The correlation between cutaneous and ocular pigmented lesions underlines the need for the adequate ophthalmological screening of subjects with CAMNs and INs.

## 1. Introduction

Recent epidemiological surveys have contributed significantly to the identification of endogenous and exogenous risk factors relating to the development of cutaneous melanocytic naevi. Despite the broadening knowledge concerning naevus and melanoma development, and prevention efforts at dermatological and national health levels, the number of individuals exhibiting large numbers of cutaneous melanocytic naevi has recently been increasing continuously. It is well established that this population is at a significantly increased of the risk of the development of both cutaneous and uveal malignant melanoma. Identification of the constitutional and environmental factors that contribute to naevogenesis is therefore an indispensable step in the primary prevention of melanoma.

In contrast with cutaneous malignant melanoma, the potential risk factors of uveal melanoma are much less clearly defined. Meta-analyses published recently have provided valuable information about the potential roles of host susceptibility factors, environmental effects and cutaneous and ocular naevi in the development of uveal melanoma [1,2,3] Few literature data are available on the endogenous and exogenous factors that may influence the formation of benign pigmented ocular lesions (ocular naevi and freckles.) [4,5] This is in striking contrast with the amount of information acquired as to the nature of cutaneous naevi. It has emerged that there is a possible relationship between CAMNs and malignant and benign uveal pigmented lesions. [6,7] There are also accumulating reports on the role of INs as uveal melanoma risk indicators.[7] Attempts to identify factors that can influence ocular naevogenesis may facilitate an understanding of the complex nature of uveal melanogenesis.

Naevogenesis is under both genetic and environmental control, and the exact roles of these factors are of special interest. Twins are classical and popular subjects in investigations of the genetic and environmental background of various disorders. Monozygotic twins have an identical genome, and dizygotic twins share almost half of their genes. The environmental exposure of twins, such as sunbathing habits, vacations in sunny climates, the use of sun creams and outdoor activities, are usually very similar, at least until adulthood, and these childhood impacts are very relevant in naevus development.

We recently conducted our first twin study, in which we identified a potential risk factor in naevus development: we observed a significantly higher prevalence of both cutaneous and uveal melanocytic naevi among twin members with a history of neonatal blue light phototherapy.[5,8,9] The aim of the present survey was to investigate the roles of simultaneous constitutional, behavioural, environmental and hereditary factors in cutaneous and uveal naevus development, and the association between these melanocytic lesions in an appreciable population of monozygotic and dizygotic twins.

## 2. Methods

### 2.1. Patients

One hundred and seventy-two pairs of twins of Caucasian origin, aged 1–34 years (mean age: 14.64 years), were included in our study, which was performed in the Department of Dermatology and Allergology and the Department of Ophthalmology at the University of Szeged. The distribution of the participating twin pairs was as follows: 61 monozygotic pairs (28 female and 33 male pairs), 44 dizygotic female pairs, 41 dizygotic male pairs, 26 dizygotic pairs of different sexes. Ophthalmological examinations could be performed in the cases of 49 monozygotic pairs (24 female and 25 male pairs) and 93 dizygotic pairs of twins (38 female, 36 male pairs and 19 pairs of different sexes) (mean age: 14.53 years). After approval and permission had been obtained from the Institutional Review Board of Albert Szent-Györgyi Medical Centre at the University of Szeged, all the participants or their parents gave their written consent before the start of the survey. The databases of the Department of Gynaecology and the Department of Paediatrics at the University of Szeged were used for recruitment of the twins.

### 2.2. Skin examinations

All twin pairs underwent a whole-body skin examination, excluding the scalp and the anogenital area. Melanocytic naevi were counted as in the standardized international protocol according to English et al. Pigmented lesions with the morphological features of CMNs, CAMNs, congenital melanocytic naevi, blue naevi, Spitz naevi, naevi spili, halo naevi, lentigines and café-au-lait macules were counted separately, and the presence of freckles was also recorded in each subject.

Skin examinations were performed by two experienced dermatologists. The validity of the naevus-counting procedure of the investigators was checked by examining several volunteers prior to the start of the survey. The agreements of the intra- and interobserver reliability, assessed by means of the Friedman ANOVA test, were found to be excellent. The skin examinations of the twin pairs were always conducted in the presence of two dermatologists; then, if any question arose, the subjects were re-examined independently by both observers.

Pigmentary traits such as eye colour and hair colour were evaluated in each subject. Eye colour was assessed on a three-category scale (1 = dark-brown, 2 = light-brown or hazel, 3 = blue, green or grey). Hair colour was classified into four categories (1 = black, 2 = brown, 3 = blond, 4 = red). Skin phototype was assessed on the Fitzpatrick scale, which is based on a person’s reaction to 30 minutes of midday sunlight for the first time in the summer (I = always burns, never tans; II = always burns, sometimes tans; III = sometimes burns, always tans; IV = never burns, always tans). Skin colour was described on a three-grade scale (dark, medium, fair).

### 2.3. Interview/questionnaire

After the clinical skin examinations, a standardized questionnaire was completed by all the participants or the accompanying parents. The questionnaire sought information on sunbathing habits (the number of severe, painful sunburns, the frequency and duration of sunbathing episodes, and the number of summer holidays besides the sea), sun protection methods used during sunbathing or various summer holidays activities, other sun exposure variables (number of days per week when more than 4 hours was spent outdoors, job, use of sunbeds and sunlamps), and a family history of a large number of melanocytic naevi, melanomas or non-melanoma skin cancers. Data relating to the neonatal history (prematurity, jaundice, and neonatal blue light phototherapy) and to the other past medical history of the subjects were also recorded.

### 2.4. Ophthalmological examination

Detailed ophthalmological examinations were carried out, including slit-lamp biomicroscopic examination of the anterior segment without dilation of the pupil (using the Inami L-0189 slit-lamp) and applanation tonometry (using the Inami L-5130 applanation tonometer). Complete indirect ophthalmoscopic examinations of the fundi were performed after maximum dilation of the pupil with cyclopentolate 0.5%, using the Heine Omega 100 indirect ophthalmoscope. All examinations were performed by a well-trained ophthalmologist.

For all participants, a standardized form was used to record the iris colour (see above), the number and distribution of IFs, INs or CHNs or any pigmented lesions of other ocular structures. Lesions identified were defined according to the Shields system of classification. [10] INs were defined as raised, discrete, pigmented lesions replacing the iris stroma and obscuring the normal iris architecture and not elevated more than 1 mm. The colourations of these lesions varied from tan to dark-brown. Lightly to darkly pigmented flat lesions on the anterior iris surface were considered to be IFs. CHNs were defined as flat to minimally elevated (not in excess of 1 mm in height) slate-grey lesions with distinct margins. The following features were noted for each CHN: basal dimensions, thickness, location, presence of drusen, subretinal fluid, orange pigment, or surrounding retinal pigment epithelial changes. Exclusion criteria were: 1) media opacity that precluded examination of the choroid, (2) iris heterochromia, (3) disorders or medication that could alter the iris colour (for example, iris neovascularization, anamnestic uveitis or ocular injury, or the use of prostaglandin analogue eyedrops), and (4) ocular or oculodermal melanocytosis or neurofibromatosis as known factors predisposing to ocular naevus formation.

### 2.5. Statistical analyses

The correlations between the prevalence of melanocytic naevi and possible endogenous and exogenous risk factors were initially assessed univariately by using the non-parametric Kruskal-Wallis test, or the Mann-Whitney test. All variables were then entered into multivariate logistic or linear regression analyses in order to evaluate the simultaneous effects of different factors on melanocytic naevus development. For pigmented cutaneous lesions, the dependent variable was the number of naevi with a logarithmic transformation; the natural logarithm of the naevus count demonstrated a normal distribution with the Kolmogorov-Smirnov one-sample test, and multivariate linear regression analysis (stepwise method) was performed. The number of BPULs did not show normal distribution by the Kolmogorov-Smirnov test and, in view of the high numbers of 0 values in the survey, multivariate logistic regression analysis (enter method) was carried out. Spearman’s rank correlation test was applied to evaluate the correlations between the numbers of CMNs, CAMNs and BPULs, with the Bonferroni correction. Interclass correlation coefficients were used to assess the relationship between cutaneous and ocular pigmented lesions among twin members. All p values calculated were two-sided, and a significance level of 0.05 was assumed. Statistical analyses were performed with SPSS version 22.0 software.

## 3. Results

### 3.1. Prevalence and risk factors of cutaneous pigmented lesions

The prevalence of CMNs and CAMNs is presented in **Tables 1 and 2** in terms of median naevus counts with interquartile ranges in different age groups.

**Table 1:** Prevalence of common and clinically atypical naevi by age groups among monozygotic twin pairs (N = 122).

Age (years)	Number of subjects	Median of number of common melanocytic naevi (CMN) (lower, upper quartile)	Median of number of clinically atypical melanocytic naevi (CAMN) (lower, upper quartile)	Median of number of melanocytic naevi (CMN+CAMN) (lower, upper quartile)
1–3	1	0 (0–0)	0 (0–0)	0 (0–0)
4–6	10	2 (1–3)	0 (0–0)	2 (1–3)
7–9	21	3 (1.5–6)	0 (0–0)	4 (1.5–6)
10–12	16	5.5 (1.25–9.75)	0 (0–0)	5.5 (1.25–9.75)
13–15	18	15 (4–28.25)	2 (0–4)	18 (4.75–33.5)
16–18	14	11.5 (3–32.5)	1 (0–7.5)	13 (3.75–43.5)
19–21	18	13 (7–21.75)	1 (0–6)	17 (7–27.75)
22–24	14	9 (4–13.5)	0 (0–1.5)	9 (4–16.5)
25–27	2	16 (8–20)	0 (0–0)	16 (8–20)
28–30	4	11 (4.75–22.5)	2 (0.25–10.5)	17.5 (7–26.5)
31–34	4	10 (4.75–10.75)	0 (0–0)	10 (4.75–10.75)

**Table 2:** Prevalence of common and clinically atypical naevi by age groups among dizygotic twin pairs (N = 222).

Age (years)	Number of subjects	Median of number of common melanocytic naevi (CMN) (lower, upper quartile)	Median of number of clinically atypical melanocytic naevi (CAMN) (lower, upper quartile)	Median of number of melanocytic naevi (CMN+CAMN) (lower, upper quartile)
1–3	4	0,5 (0–1.75)	0 (0–0)	0.5 (0–1.75)
4–6	48	1 (0–3)	1 (0–0)	1 (0–3)
7–9	26	6 (1.75–10.25)	0 (0–1)	6.5 (1.75–12.25)
10–12	32	6 (1–9)	0 (0–0)	6 (1–9.75)
13–15	22	12 (3–20.5)	1 (0–2.25)	13 (4.75–21.5)
16–18	20	16 (5.75–22.75)	0.5 (0–2.75)	16.5 (5.75–29.5)
19–21	16	12 (4.5–35.75)	1.5 (0–2.75)	13 (6–40)
22–24	26	16.5 (4.75–31.75)	1 (0–4)	19 (5–36.25)
25–27	10	12.5 (7.5–23.75)	1 (0–2.5)	14 (8–25.75)
28–30	12	23 (15.75–35.25)	0.5 (0–2.75)	23.5 (17.5–37.5)
31–34	6	16.5 (5.5–42)	0.5 (0–2.75)	19 (6.25–43.5)

The interclass correlation coefficients for CMN, CAMN and total naevus count were 0.77, 0.76 and 0.81 in monozygotic twins, as compared with 0.5, 0.27 and 0.5 in dizygotic twin pairs, respectively.

Statistically significant associations were observed between the density of naevi (CMNs+CAMNs) and eye colour, hair colour, skin phototype, the frequency and duration of use of sunscreens, a history of severe sunburns during childhood and adolescence, the frequency and duration of sunbathing, more time spent outdoors during childhood, an outdoor job, the number of summer holidays beside the sea in the Mediterranean, or in a subtropical or a tropical climate, the use of sunbeds and sunlamps, a history of large numbers of naevi among siblings, and a history of neonatal blue light phototherapy with the Mann-Whitney and the Kruskall-Wallis tests. When the analysis was focused separately on the risk factors of CMNs and CAMNs, the same factors proved to be influence the number of CMNs. A history of severe painful sunburns during childhood, the frequency and duration of sunbathing, the use of sunbeds and sunlamps, the number of summer holidays beside the sea in the Mediterranean, or in a subtropical or a tropical climate, an outdoor job, and a history of large numbers of naevi among siblings were significantly associated with the prevalence of CAMNs (Table 3 and S1 Table)

**Table 3:** Association between gender, constitutional and sun exposure variables and the prevalence of melanocytic naevi among monozygotic (N = 122) and dizygotic (N = 222) twin pairs.

Factors	Monozygotic twin pairs			Dizygotic twin pairs			
	N	Quartiles	P	N	Quartiles	P	
**Gender**							
male	66	6.5 (3–17.25)		108	6 (1–16.75)		
female	56	8 (3.25–20.5)	0.859	114	8 (2–20)	0.273	a
**Eye colour**							
brown	57	4 (2–10.5)		101	6 (1–17.5)		
hazel, greenish brown	13	10 (5.5–17)		22	8.5 (4–22.25)		
green, grey, blue	52	10.5 (4–25.75)	0.040*	99	8 (2–21)	0.314	b
**Hair colour**							
black, dark-brown	19	3 (2–16)		50	13 (2.75–25.75)		
medium-brown, light-brown	97	8 (3–17)		146	6 (2–18.25)		
blond	6	21.5 (2.75–64.25)	0.198	26	2.5 (1–12)	0.040*	**b**
**Skin colour**							
dark, medium	76	6.5 (2–13.75)		127	8 (2–19)		
fair	46	9 (3.75–25.5)	0.071	93	7 (2–18)	0.767	a
**Skin phototype**							
I-II	30	10.5 (6–27.5)		46	9.5 (2–24)		
III IV	92	6 (2–13.75)	0.001*	176	6 (1–18.75)	0.087	a
**Frequency of use sunscreen**							
never	19	11 (4–30)		17	7 (1–29)		
occasionally	46	7.5 (3–18.25)		74	12 (4–24)		
always at the beginning of the summer, then occasionally	29	9 (2–24)		61	6 (1–13.5)		
regularly	28	4 (2.25–8.75)	0.208	70	5 (2–14)	0.011*	**b**
**SPF**							
0	18	10.5 (3.5–19.5)		18	7.5 (1–25.5)		
1–10	8	6.5 (0.75–23.25)		23	12 (6–24)		
10–20	43	8 (3–15)		69	12 (4–26.5)		
>20	50	6 (3–22.5)	0.758	111	4 (1–12)	0.000*	**b**
**Duration of use of sunscreen**							
never	25	10 (2.5–26)		34	7.5 (1.75–23.25)		
1–5 years	45	7 (2–12.5)		82	3 (1–10)		
6–10 years	27	4 (2–9)		53	9 (2.5–19.5)		
10–20 years	23	21 (6–39)	0.002*	51	14 (5–27)	0.000*	**b**
**Number of severe painful sunburns during childhood**							
0	79	6 (2–11)		131	5 (1–15)		
1–2	29	14 (4–27.5)		67	8 (2–17)		
3–5	14	12 (2–30)	0.010*	22	19.5 (8.75–42.5)	0.000*	**b**
**Number of severe painful sunburns during adolescence**							
0	53	9 (4–22)		71	12 (4–21)		
1–2	21	22 (4.5–39)		43	23 (9–35)		
3–5	6	11 (7.5–26.25)	0,281	8	33 (10–56.75)	0.006*	**b**
**Number of severe painful sunburns during adulthood**							
0	35	10 (4–22)		47	14 (5–33)		
1–5	12	11 (8.25–26.5)	0.558	28	16.5 (8–39.75)	0.405	a
**Frequency of sunbathing between April and September**							
0	16	9.5 (3–29.75)		61	4 (1–17.5)		
1–10	38	8.5 (4.75–21.25)		48	8.5 (3–23.75)		
10–20	17	22 (5.5–39)		38	8.5 (1–22)		
>20	49	4 (2–9.5)	0.000*	67	9 (4–18)	0.173	b
**Duration of one sunbathing episode**							
<half hour	18	3.5 (1.75–17.25)		53	7 (1.5–17.5)		
half hour—1 hour	33	10 (4.5–21.5)		54	12 (4–31)		
1–3 hours	44	6.5 (3–24.25)		61	6 (1–17)		
>3 hours	17	3 (1–8.5)	0.047*	27	10 (6–18)	0.024*	**b**
**Number of days per week when more than 4 hours was spent outdoors during childhood**							
0–1	14	14.5 (6–23.25)		29	4 (1–10.5)		
2–3	48	9 (4–25)		57	9 (3–21.5)		
4–5	33	9 (2–15)		83	8 (2–20)		
6–7	27	3 (2–7)	0.023*	53	7 (1–19)	0.157	b
**Number of days per week when more than 4 hours was spent outdoors during adolescence**							
0–1	11	21 (4–27)		19	12 (2–24)		
2–3	38	11 (6.25–30)		46	15.5 (7.5–31)		
4–5	16	11 (3–17)		43	14 (7–35)		
6–7	17	7 (3.5–23.5)	0.382	11	19 (2–36)	0.356	b
**Number of days per week when more than 4 hours was spent outdoors during adulthood**							
0–1	13	21 (6.5–48)		20	23 (6.5–31)		
2–3	14	10.5 (8–26.25)		27	14 (7–37)		
4–5	10	12.5 (3.75–21.25)		13	14 (8–29.5)		
6–7	9	8 (5–17.5)	0.434	14	28.5 (3.75–44.25)	0.885	b
**Employment**							
interior	53	8 (4–26.5)		95	12 (5–24)		
interior and outdoor	46	9 (3–13.25)		72	5 (1.25–13)		
outdoor	9	8 (6–24)	0.475	8	42.5(12.25–46.75)	0.000*	b
**Number of summer holiday beside the sea in the Mediterranean or in s subtropical climate**							
0	66	9 (3–16)		111	4 (1–13)		
1–2	28	4 (2.25–16.25)		59	6 (1–17)		
3–4	16	8 (0.75–22.5)		18	14 (4–31.5)		
> = 5	12	16 (4.5–31.25)	0.240	34	18 (8.75–28.5)	0.000*	b
**Use of sunbeds**							
never	101	7 (2–15.5)		201	6 (1.5–17.5)		
occasionally, regularly	21	10 (5–28.5)	0.032*	21	14 (7–32)	0.012*	a
**Family history of large numbers of melanocytic naevi (parent)**							
yes	48	6.5 (3–25.75)		90	9 (2–23)		
no	56	10 (4–20.25)	0.271	104	6 (2–17)	0.139	a
**Family history of large numbers of melanocytic naevi (sibling)**							
yes	22	27.5 (7–46)		46	13 (5–28.5)		
no	76	7 (3–12.75)	0.000*	148	5 (1–14)	0.000*	a
**Family history of malignant melanoma (parent)**							
yes	0	-		0	-		
no	108	8 (3–18.75)	-	208	7 (2–18.5)	-	
**Family history of malignant melanoma (sibling)**							
yes	0	-		0	-		
no	106	8 (3–16.5)	-	206	7 (2–19)	-	
**Family history of malignant melanoma (grandparent)**							
yes	4	15 (1.75–51.5)		4	12 (6.25–29.75)		
no	90	7 (3–14)	0.623	184	6 (2–19)	0.286	a
**Neonatal blue light phototherapy**							
yes	69	11 (3.5–25)		120	9 (3.25–20.75)		
no	53	6 (2–11)	0.026*	100	5 (1–15)	0.020*	a

a: Mann-Whitney test

b: Kruskal-Wallis test

*: p<0,05

### 3.2. Prevalence and risk factors of benign ocular pigmented lesions

The prevalence of BPULs in our study population was 31.4%. 25.35% of the subjects had at least one IF, INs were detected in 17 persons (5.98%), and CHNs were seen in 10 persons (3.52%) (Table 4 and S1 Table).

**Table 4:** Prevalence of benign pigmented ocular lesions among monozygotic and dizygotic twin pairs.

	Monozygotic pairs (98 person)		Dizygotic pairs (186 person)	
	Number of subjects with lesions	Number of lesions	Number of subjects with lesions	Number of lesions
Iris naevus	7	9	10	14
Choroidal naevus	7	7	3	4
Iris freckles	28	129	44	215

The interclass correlation coefficients for IF, IN, CHN count and the total number of BUPLs were 0.42, 0.86, 0.38 and 0.41 in monozygotic twins, as compared with 0.38, 0.25, 0.02 and 0.35 in dizygotic twin pairs, respectively.

On univariate analysis, a lighter eye colour, a history of severe sunburns in childhood, the frequency of sunbathing, more time spent outdoors during adulthood, the family history of large numbers of cutaneous melanocytic naevi and a history of neonatal blue light phototherapy proved to be associated with a substantially higher prevalence of benign ocular pigmented lesions. On multivariate logistic regression analysis, the age and the frequency of sunbathing were significantly related to the density of BPULs. The strongest risk factor of BPULs was neonatal blue light phototherapy, which resulted in a relative risk of 4.88 for the development of BPULs (Table 5 and S1 Table).

**Table 5:** Association between gender, constitutional and sun exposure variables and the prevalence of benign ocular pigmented lesions among monozygotic (98 persons) and dizygotic (186 persons) twin pairs.

Factors	Monozygotic twin pairs		Dizygotic twin pairs		

	N/person	P	N/person	P	
**Gender**					
male	83/50		133/91		
female	62/48	0.363	100/95	0.333	a
**Eye colour**					
brown	68/43		113/81		
hazel, greenish-brown	36/9		50/21		
green, grey, blue	41/46	0.004*	70/84	0.133	b
**Hair colour**					
black, dark-brown	17/15		60/44		
medium-brown, light-brown	127/81		153/121		
blond	1/2	0.660	20/21	0.442	b
**Skin colour**					
dark, medium	79/65		150/106		
fair	66/33	0.632	77/78	0.062	a
**Skin phototype**					
I-II	58/20		44/39		
III-IV	87/78	0.099	189/147	0.170	a
**Number of severe painful sunburns during childhood**					
0	94/65		89/105		
1–2	26/22		113/59		
3–5	25/11	0.611	27/20	0.004*	b
**Number of severe painful sunburns during adolescence**					
0	58/41		95/50		
1–2	32/18		55/36		
3–5	1/3	0.278	5/8	0.629	b
**Number of severe painful sunburns during adulthood**					
0	38/27		59/34		
1–2	3/8		12/19		
3–5	1/1	0.802	13/6	0.085	b
**Frequency of sunbathing between April and September**					
0	15/14		27/56		
1–10	57/34		41/41		
10–20	8/12		72/30		
>20	63/36	0.257	86/51	0.004*	b
**Duration of one sunbathing episode**					
< half hour	32/15		61/48		
half hour—1 hour	32/26		67/47		
1–3 hours	48/37		72/46		
> 3 hours	27/10	0.435	16/20	0.412	b
**Number of days per week when more than 4 hours was spent outdoors during childhood**					
0–1	20/12		22/23		
2–3	69/38		62/45		
4–5	22/25		97/72		
6–7	34/23	0.512	52/46	0.683	b
**Number of days per week when more than 4 hours was spent outdoors during adolescence**					
0–1	13/8		42623		
2–3	56/28		42/39		
4–5	4/13		79/35		
6–7	21/15	0.074	24/8	0.146	b
**Number of days per week when more than 4 hours was spent outdoors during adulthood**					
0–1	13/8		10/5		
2–3	17/10		37/19		
4–5	0/9		8/11		
6–7	12/9	0.029*	31/14	0.092	b
**Employment**					
interior	69/44		92/74		
interior and outdoor	37/35		92/64		
outdoor	5/9	0.440	12/8	0.258	b
**Number of summer holiday beside the sea in the Mediterranean, or in a subtropical or tropical climate**					
0	92/56		121/99		
1–2	24/21		52/46		
3–5	29/21	0.738	60/41	0.801	b
**Use of sunbeds**					
never	126/82		206/168		
occasionally, regularly	19/16	0.574	27/18	0.281	a
**Family history of large numbers of melanocytic naevi (parent)**					
yes	67/42		102/78		
no	65/42	0.583	108/84	0.862	a
**Family history of large numbers of melanocytic naevi (sibling)**					
yes	45/16		31/38		
no	81/62	0.011*	183/124	0.480	a
**Family history of malignant melanoma (parent)**					
yes	0/0		0/0		
no	125/84	-	215/174	-	
**Family history of malignant melanoma (sibling)**					
yes	0/0		0/0		
no	125/82	-	211/172	-	
**Family history of malignant melanoma (grandparent)**					
yes	0/2		0/4		
no	112/72	0.403	190/154	0.239	a
**Neonatal blue light phototherapy**					
yes	120/52		154/98		
no	25/46	0.000*	76/86	0.035*	a

a: Mann-Whitney test

b: Kruskal-Wallis test

*: p<0,05

### 3.3. Association between cutaneous and ocular pigmented lesions

The number of CMNs was strongly associated with the number of CAMNs and the density of lentigines. A statistically significant correlation was found between the prevalence of CAMNs and INs. Similar tendencies were observed in the monozygotic and dizygotic subgroups. There were significant associations between the prevalence of CAMNs and the numbers of INs, IFs, and all BUPLs in the dizygotic twins.

## 4. Discussion

During the past few decades, the incidence of malignant melanoma has been continuously and rapidly increasing, particularly among Caucasian populations. Various genetic, constitutional and environmental factors that may contribute to melanoma development have been objects of intensive scientific investigations for a long time, in view of the very aggressive biological behaviour of this tumour type. It is well established that the presence of large numbers of CMNs and CAMNs is the most important independent phenotypic marker of the development of melanoma. [11] The results of epidemiological surveys relating to risk factors for naevus development as concerns pigmentary traits, skin phototype, sunbathing habits and sun protection methods, and other sun exposure variables are conflicting. The inconsistencies may stem from the fact that these surveys were conducted on different populations and different age groups. In the present study, a fair complexion, i.e. a lighter eye colour and skin phototype I-II. was associated with a higher prevalence of naevi. It is well established that both acute, intense, intermittent and chronic, not continuous UV exposure play important roles in naevogenesis. Accordingly, severe, painful sunburn, summer holidays in sunny climates, frequent and long sunbathing, frequent use of sunlamps, and outdoor occupational activities all result in a significantly elevated number of cutaneous melanocytic naevi. Our results also underline the role of a special, recently identified risk factor in naevus development; neonatal blue light phototherapy as acute, intense exposure may have a considerable impact on the immature melanocytes, and can induce naevogenesis.

In contrast with cutaneous melanoma, the incidence of uveal melanoma has remained relatively stable in recent decades, but, despite advances in treatment modalities, the survival rate in cases of uveal melanoma has not improved [12,13,14] The early detection of the disease is therefore essential, as each millimetre increase in uveal melanoma thickness leads to a 5% increased risk of metastasis by 10 years [15] The identification of factors that may contribute to the development of uveal melanoma is of great importance from the aspect of the possibility of earlier diagnosis and treatment. Evidence is accumulating that, besides the presence of CAMNs, INs can also serve as a marker of a predisposed phenotype at risk of uveal melanoma. [7] Despite the potential marker role in melanogenesis, the nature of ocular naevus formation is considerably underinvestigated. The aim of our present investigation was to examine factors involved in ocular naevogenesis. We categorized our results and sought conclusions in four conceptual subgroups: epidemiology; host factors; external exposure (UV radiation and neonatal blue light phototherapy); and associations with cutaneous naevi. Presence of cutaneous naevi unquestionably represent combined effects of host susceptibility factors and environmental impacts.

There have been few epidemiologic surveys of the incidence of ocular naevi among Caucasians. INs have been reported to occur in 4–6% of the white population, whereas IFs are considerably more frequent, with an estimated incidence of 25–60%. [16] The reported incidence of CHNs ranges widely from 0.2 to 18% [16,17,18] The largest population-based study, the Blue Mountains Eye Study led to an incidence of CHNs of 6.5% in a white population. [18] The wide range of variation may be due to the differences in the study populations (differences in eye colour distribution, age etc.). Approximately one-third (31.4%) of the participants in our cohort had one or more BPULs, while the incidence of IFs, INs and CHNs proved to be 25.35%, 5.98% and 3.52%, respectively. In our earlier twin study of a substantially smaller cohort, the number of melanocytic lesions of the iris proved to be age- independent, while in our present study, the incidence of these lesions increased with advancing age. Ocular naevi are rarely identified in infants, but they begin to be detected in the first decade of life. [19] This could mean that naevi are not present at birth or that they become sufficiently pigmented to be detectable only at a later age. Among the host susceptibility factors that we examined, a lighter iris colour proved to be positively associated with the prevalence of BPULs. We did not find a correlation with hair the colour, the skin phototype or the density of BPULs. A light iris colour is one of the most consistent risk factors for uveal melanoma. In the meta-analysis by Weis et al. most of the published studies detected a significant correlation of the uveal melanoma risk with a light iris colour. [2]

Among the UV exposure variables, a history of severe sunburns in childhood, the frequency of sunbathing, and more time spent outdoors during adulthood proved to be positively correlated with the frequency of BPULs. We are not aware of any earlier investigation regarding the connection between UV radiation and ocular naevus formation. The role of UV radiation in the risk of uveal melanoma is controversial: the results of meta-analyses of published reports are inconsistent. [20,21] Artificial UV radiation seems to be a possible risk factor. [22,23]

We previously observed significantly elevated numbers of IFs in cases with anamnestic neonatal blue light phototherapy.[5] Our present results are in accordance with the results of our earlier twin study on a smaller cohort. The light transmissibility profile of the ocular media may allow the penetration of an appreciable amount of potentially harmful light into the eye. Further studies are needed to clarify the potential long-term effects of neonatal blue light phototherapy on melanocytic proliferation of the uveal tract.

A lighter eye colour, a history of severe, painful sunburns, the frequency of summer holidays, and a history of neonatal blue light phototherapy proved to be common risk factors for both cutaneous and ocular melanocytic lesions. Although the biological behaviour of uveal and cutaneous melanocytes is markedly different, their relationship is based on the fact that these dendritic cells share a common neuroectodermal origin: they both arise from the embryological neural crest, and then migrate and reach their respective sites during embryologicalal development. [24,25] We earlier found a substantially higher prevalence of BPULs in patients with cutaneous atypical naevi. [5] In another study, we observed an elevated number of cutaneous dysplastic naevi in subjects with uveal melanoma.[4] The recently published meta-analysis by Weis et al. strengthens the view of associations between CMNs, CAMNs, INs and an elevated prevalence of uveal melanoma.^7^ Our present results reveal statistically significant correlations between the densities of CMNs and CAMNs and the number of INs in the monozygotic twin group. As concerns the dizygotic twins, positive associations were found between the prevalence of CAMNs and the presence of INs and IFs.

There do not appear to be any earlier reports of the interclass correlation of ocular naevi in monozygotic and in dizygotic twins, although such a statistical parameter can provide valuable data about the genetic influence on naevogenesis. The interclass correlation coefficient of 0.86 for INs among monozygotic twins indicates a comparatively strong genetic determination of these pigmented lesions, in contrast with IFs or CHNs. This finding is especially interesting in light of the presumed role of INs as a phenotypic risk indicator for uveal melanoma.

The similar genetic background of twins and the similar environmental impacts to which they are exposed allow an exact and objective investigation of different exogenous and endogenous factors in naevus development. So far there have been few twin studies on the prevalence and risk factors of melanocytic naevi, mainly investigating the relative contribution of heredity and environment to naevus number [26,27,28,29] and this appears to be the first survey in which cutaneous and ocular pigmented lesions are considered simultaneously in a relatively large sample of identical and non-identical twins. The significant correlation that we detected between the INs and CAMNs suggests that a subgroup of the Caucasian population may be particularly susceptible to both cutaneous and ocular naevus formation. Multifactorial effects, including the genetic background, influenced by environmental insults, may have roles in this process. The regular dermatological screening of individuals with large numbers of CAMNs may facilitate the early recognition and successful treatment of malignant melanoma. The correlation between cutaneous and ocular pigmented lesions highlights the need for the adequate ophthalmological screening of subjects with CAMNs.

We plan to investigate our twin population at 5-year intervals in order to acquire valuable information concerning the time course of ocular and cutaneous naevus formation. Further studies are necessary to clarify the exact processes and the relationship between uveal and cutaneous naevus development.

## Supporting Information

S1 Table All data from the examined twin pairs can be found in the Supporting Table. (XLS)

## Abbreviations

CAMN: Clinically atypical melanocytic naevus
BUPL: Benign uveal pigmented lesion
IF: Iris freckle
IN: Iris naevus
CHN: Choroidal naevus
CMN: Common melanocytic naevus
