# Correction: Risk Factors and Relationship of Cutaneous and Uveal Melanocytic Lesions in Monozygotic and Dizygotic Twin Pairs

**DOI:** 10.1371/journal.pone.0163189

**Published:** 2016-09-14

**Authors:** Renáta Zsanett Csoma, Edit Tóth-Molnár, Anita Varga, Hajnalka Szabó, Hajnalka Orvos, Lajos Kemény, Judit Oláh

Affiliation 5 appears incorrectly in the published article. Affiliation 5 should be: MTA-SZTE Dermatological Research Group, Szeged, Hungary.

The affiliations for the sixth author are incorrect. Lajos Kemény is affiliated with number 1: Department of Dermatology and Allergology, University of Szeged, Szeged, Hungary, and with number 5: MTA-SZTE Dermatological Research Group, Szeged, Hungary.
